# Poorer breast cancer survival outcomes in males than females might be attributable to tumor subtype

**DOI:** 10.18632/oncotarget.12052

**Published:** 2016-09-15

**Authors:** Qi Wu, Juanjuan Li, Shan Zhu, Juan Wu, Xiang Li, Qian Liu, Wen Wei, Shengrong Sun

**Affiliations:** ^1^ Department of Breast and Thyroid Surgery, Renmin Hospital of Wuhan University, Wuhan, Hubei, P. R. China; ^2^ Department of Pathology, Renmin Hospital of Wuhan University, Wuhan, Hubei, P. R. China; ^3^ Department of Breast Surgery, Hubei Cancer Hospital, Wuhan, Hubei, P. R. China

**Keywords:** breast cancer, male breast cancer, SEER data

## Abstract

**Background & Aims:**

Substantial controversy exists regarding the differences in tumor subtypes between male breast cancer (MBC) and female breast cancer (FBC). This is the largest population-based study to compare MBC and FBC patients.

**Methods:**

Using data obtained by the Surveillance, Epidemiology, and End Results (SEER) program from 2010-2012, a retrospective, population-based cohort study was conducted to investigate tumor subtype-specific differences in various characteristics, overall survival (OS) and breast cancer-specific mortality (BCSM) between males and females.

**Results:**

In all, 181,814 BC patients (1,516 male and 180,298 female) were eligible for this study. The male patients were more likely to be black, older, and have lower histological grades, more advanced stages, larger tumors, more lymph node and distant metastases and human epidermal growth factor receptor 2 (HER2)-negative tumors (each *p*<0.05). A matched analysis showed that the 2-year OS was 91.2% and 93.7% and that the BCSM was 2.2% and 2.5% for male and female patients, respectively. The univariate analysis showed that male triple-negative (TN), hormone receptor (HoR)-positive/HER2-positive and HoR-positive/HER2-negative patients had poorer OS (*p* <0.01). Meanwhile, the HoR-positive/HER2-positive and TN subtypes were associated with a higher BCSM in MBC patients (*p*<0.01). The multivariate analysis revealed that TN MBC patients had poorer OS and BCSM (*p*<0.05). Simultaneously, the results showed that male patients in the HoR-positive/HER2-negative subgroup were less likely to die of BC when adjusting for other factors (*p*<0.05).

**Conclusions:**

The analysis of 2-year OS and BCSM among the BC subtypes showed clear differences between MBC and FBC patients with the TN subtype; these differences warrant further investigation

## INTRODUCTION

Male breast cancer (MBC) is an uncommon disease, constituting less than 1% of all BCs and approximately 0.2% of all male cancers [1, 2]. In contrast to the incidence of female breast cancer (FBC), the incidence of MBC has been steadily increasing over the past 3 decades [3–5]. The low incidence of the disease has resulted in only a superficial knowledge of its etiology, biological behavior, and treatment. The prognostic factors, epidemiological factors, and behavior of BC are different for males than females [3]. As more data on the tumor biology of MBC emerges, it is becoming clear that MBC is a unique disease requiring its own trials and treatment guidelines.

Although the mortality and survival rates have been significantly improved for both MBC and FBC patients, more progress has been made in FBC than MBC [6, 7]. Because of the rarity of MBC, no randomized trials have been possible, and most information is derived from retrospective studies. Several studies have compared MBC and FBC and have found that MBC was not only associated with a worse prognosis but also showed more advanced stages and older ages at diagnosis, inappropriate staging, increased comorbidities, and more aggressive tumor biology [8–10]. Other studies have shown no association between MBC and survival and have suggested that patient sex does not influence mortality [11–13]. Thus, there is no consensus on the relationship between sex and prognosis in BC. Given that treatment strategy and patient management depend on prognostic variables, we used data from the National Cancer Institute's Surveillance, Epidemiology, and End Results (SEER) program to analyze the association of each tumor subtype with overall survival (OS) and breast cancer-specific mortality (BCSM) at diagnosis for males and females diagnosed with BC.

## RESULTS

### Patient characteristics

A total of 1,516 MBC and 180,298 FBC patients were eligible during the 2010-2012 study period. We excluded 710 patients whose survival times were classified as unknown from the analysis. A total of 1,339 MBC and 162,448 FBC patients had tumor subtype information available and were included in this study.

Differences in patient demographics, cancer characteristics, treatments, and outcomes between the two groups are summarized in Table 1. In all, 59.5% of the MBC patients were ≥ 65 years old at the time of diagnosis. Males were more likely to be black and at more advanced stages (each *p* < 0.01). Biological tumor characteristics also differed significantly between the sexes. MBC was more likely to be lower in grade, larger in size, have more lymph node and distant metastases and be human epidermal growth factor receptor 2 (HER2) negative (each *p* < 0.05). Males were also less likely to receive radiation than females (*p* < 0.01).

**Table 1:** Characteristics of male and matched female patients with breast cancer

Variables	Female *N* = 180298(%)	Male*N* = 1516(%)	*P*
**Follow-up time, months**	15.88±10.34	15.5±10.44	
**Age at diagnosis, years**			< 0.01
**<35**	3329(1.8)	12(0.8)	
**35-49**	33520(18.6)	147(9.7)	
**50-64**	67645(37.5)	455(30.0)	
**≥65**	75804(42.1)	902(59.5)	
**Race**			< 0.01
**white**	143614(79.7)	1212(79.9)	
**Black**	19763(11.0)	213(14.1)	
**Other**	15432(8.6)	78(5.1)	
**Unknown**	1489(0.8)	13(0.9)	
**Grade**			< 0.01
**Well**	38292(21.2)	179(11.8)	
**Moderately**	73061(40.5)	713(47.0)	
**Poorly**	54471(30.2)	501(33.0)	
**Undifferentiated**	905(0.5)	4(0.3)	
**Unknown**	13569(7.5)	119(7.8)	
**Stage**			< 0.01
**I**	98878(54.8)	632(41.8)	
**II**	55199(30.6)	560(36.9)	
**III**	18995(10.5)	252(16.6)	
**IV**	7006(3.9)	72(4.7)	
**Unknown**	220(0.1)	-	
**Tumor size**			< 0.01
**T0/T1**	103334(57.3)	694(45.8)	
**T2**	50479(28.0)	566(37.3)	
**T3**	18047(10.0)	42(2.7)	
**T4**	8218(4.6)	130(8.5)	
**NA**	220(0.1)	84(5.5)	
**Node stage**			< 0.01
**N0**	119544(66.3)	836(55.1)	
**N1**	39702(22.0)	434(28.6)	
**N2**	9384(5.2)	131(8.6)	
**N3**	6113(3.4)	72(4.7)	
**NX**	5335(3.0)	43(2.8)	
**NA**	220(0.1)	-	
**Distant metastasis**			0.02
**M0**	170033(94.3)	1411(93.1)	
**M1**	10045(5.6)	105(6.9)	
**NA**	220(0.1)	-	
**Laterality**			0.11
**Left**	90962(50.5)	797(52.6)	
**Right**	88083(48.9)	703(46.4)	
**Paired**	1046(0.6)	12(0.8)	
**Bilateral**	104(0.1)	-	
**Unknown**	103(0.1)	4(0.3)	
**HER2**			< 0.01
**Negative**	138954(77.1)	1191(78.6)	
**Positive**	23990(13.3)	153(10.1)	
**Borderline**	4194(2.3)	47(3.1)	
**Unknown**	13160(7.3)	125(8.2)	
**Radiotherapy**			< 0.01
**No**	92832(51.5)	1109(73.2)	
**Yes**	86039(47.7)	396(26.1)	
**Unknown**	1427(0.8)	11(0.7)	
**Status**			< 0.01
**Alive**	168929(93.7)	1382(91.2)	
**Dead**	11369(6.3)	134(8.8)	

y years

### Impact of tumor subtype on OS in MBC and FBC

**Figure 1:**
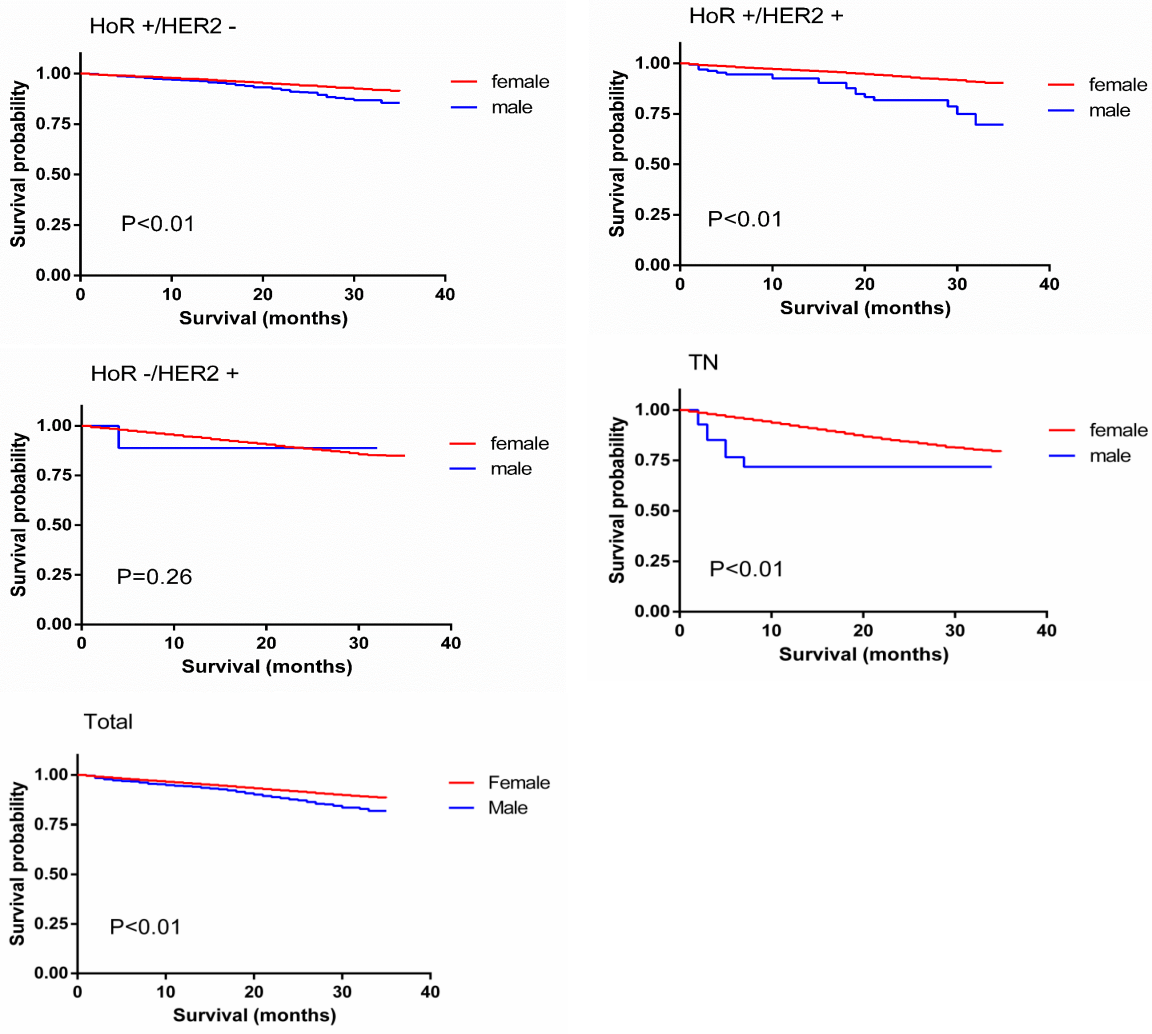
Overall survival curves of male matched with female breast cancer patients

A Kaplan-Meier analysis was used to determine OS in the groups based on sex. Individual survival curves for the four subgroups were generated (Figure 1). As expected, male patients exhibited poorer survival rates than female patients (*p* < 0.01). Unexpectedly, the stratified analysis indicated that the survival rates were similar for MBC and FBC patients in the hormone receptor (HoR)-negative/HER2-positive group, whereas in the other groups, OS was better for FBC patients than MBC patients (*p* < 0.01).

We performed univariate and multivariate analyses based on the Kaplan-Meier results. In the univariate analysis, age at diagnosis, sex, race, tumor grade, laterality, tumor stage, tumor size, node stage, distant metastasis, HER2 status and history of radiation were all significantly associated with OS (*p* < 0.05). A multivariate analysis using the Cox regression model was also performed. All the factors mentioned above were identified as independent prognostic factors (Table 2), including age at diagnosis (50-64, hazard ratio (HR) = 1.678 (1.391, 2.024); ≥65, HR = 3.772 (3.133, 4.541)), sex (HR = 0.923 (0.778,1.095)), race (black, HR = 1.34 (1.275, 1.408)), grade (moderately differentiated, HR = 1.139 (1.062, 1.221); poorly differentiated, HR = 1.902 (1.774, 2.04); undifferentiated, HR = 2.037 (1.673, 2.48)), tumor stage (II, HR = 1.134 (1.047, 1.229); III, HR = 2.862 (2.606, 3.143); IV, HR = 2.405 (2.165, 2.672)), tumor size (T2, HR = 1.628 (1.513, 1.752); T3, HR = 2.296 (2.134, 2.47); T4, HR = 1.875 (1.712, 2.053)), node stage (N1, HR = 1.133 (1.078, 1.919); N2, HR = 1.183 (1.093, 1.28); N3, HR = 1.498 (1.397, 1.607)), distant metastasis (M1, HR = 2.857 (2.668, 3.06)), laterality (bilateral, HR = 1.167 (1.053, 1.294)), HER2 status (positive, HR = 1.507 (1.425, 1.594); borderline, HR = 1.09 (0.977, 1.217)) and history of radiation (no, HR = 1.124 (0.968, 1.306)).

**Table 2:** Cox proportional hazards regression model analysis of overall survival

Variables	Univariate analysis		Multivariate analysis	
	HR (95% CI)	*P*-value	HR (95% CI)	*P*-value
**Age at diagnosis, y**				
**<35**	Reference		Reference	
**35-49**	0.92(0.759,1.116)	0.399	1.174(0.968,1.424)	0.104
**50-64**	1.245(1.032,1.501)	0.022	1.678(1.391,2.024)	< 0.001
**≥65**	2.859(2.376,3.44)	< 0.001	3.772(3.133,4.541)	< 0.001
**Sex**	1.431(1.207,1.697)	< 0.001	0.923(0.778,1.095)	0.359
**Race**				
**white**	Reference		Reference	
**Black**	1.631(1.553,1.713)	< 0.001	1.34(1.275,1.408)	< 0.001
**Other**	0.665(0.613,0.721)	< 0.001	0.753(0.695,0.817)	< 0.001
**Unknown**	0.61(0.452,0.823)	0.001	0.456(0.338,0.616)	< 0.001
**Grade**				
**Well**	Reference		Reference	
**Moderately**	1.519(1.418,1.627)	< 0.001	1.139(1.062,1.221)	< 0.001
**Poorly**	2.931(2.743,3.132)	< 0.001	1.902(1.774,2.04)	< 0.001
**Undifferentiated**	4.541(3.744,5.507)	< 0.001	2.037(1.673,2.48)	< 0.001
**Unknown**	8.102(7.551,8.692)	< 0.001	1,821(1.682,1.972)	< 0.001
**Stage**				
**I**	Reference		Reference	
**II**	1.835(1.734,1.942)	< 0.001	1.134(1.047,1.229)	0.002
**III**	3.422(2.788,4.088)	< 0.001	2.862(2.606,3.143)	< 0.001
**IV**	9.755(9.133,10.419)	< 0.001	2.405(2.165,2.672)	< 0.001
**Unknown**	9.757(7.129,12.859)	< 0.001	4.822(3.565,6.521)	< 0.001
**Tumor size**				
**T0/T1**	Reference		Reference	
**T2**	2.462(2.339,2.592)	< 0.001	1.628(1.513,1.752)	< 0.001
**T3**	7.843(7.454,8.252)	< 0.001	2.296(2.134,2.47)	< 0.001
**T4**	8.863(6.601,11.90)	< 0.001	1.875(1.712,2.053)	< 0.001
**Node stage**				
**N0**	Reference		Reference	
**N1**	2.013(1.924,2.107)	< 0.001	1.133(1.078,1.919)	< 0.001
**N2**	2.367(2.202,2.544)	< 0.001	1.183(1.093,1.28)	< 0.001
**N3**	4.397(4.109,4.706)	< 0.001	1.498(1.397,1.607)	< 0.001
**NX**	5.844(4.357,7.838)	< 0.001	1.06(0.978,1.15)	0.156
**Distant metastasis**				
**M0**	Reference		Reference	
**M1**	5.158(3.848,6.914)	< 0.001	2.857(2.668,3.06)	< 0.001
**Laterality**				
**Left**	Reference		Reference	
**Right**	0.961(0.925,0.997)	0.145	0.953(0.918,0.989)	0.102
**Paired**	1.163(1.126,1.297)	0.146	0.905(0.662,1.236)	0.530
**Bilateral**	2.332(2.105,2.371)	< 0.001	1.167(1.053,1.294)	0.003
**Unknown**	1.206(0.856,1.698)	0.285	1.064(0.776,1.457)	0.701
**HER2**				
**Negative**	Reference		Reference	
**Positive**	3.635(3.471,3.807)	< 0.001	1.507(1.425,1.594)	< 0.001
**Borderline**	1.464(1.312,1.634)	< 0.001	1.09(0.977,1.217)	< 0.001
**Unknown**	1.178(1.114,1.246)	< 0.001	0.832(0.786,0.881)	0.123
**Radiotherapy**				
**Yes**	Reference		Reference	
**No**	1.908(1.6472.211)	< 0.001	1.124(0.968,1.306)	< 0.001

HER2 human epidermal growth factor receptor 2; HoR hormone receptor;

Table 3 shows a summary of OS in MBC and FBC as determined by the subgroup analysis. After a median follow-up period of 15 months (range, 0-35 months), 91.2% of men and 93.7% of women were alive in the overall cohort. The univariate analysis of OS according to tumor subtype showed significant differences between MBC and FBC; lower survival was observed in males than females with the TN (2-year OS: 77.4%, *p* < 0.01), HoR-positive/HER2-positive (2-year OS: 85.8%, *p* < 0.01) and HoR-positive/HER2-negative subtypes (2-year OS: 93.8%, *p* < 0.01). The results of the multivariate analysis were somewhat inconsistent with these findings, as they showed decreased survival in males only for the TN subtype (HR = 2.251 (1.058, 4.787), *p* < 0.05).

**Table 3:** Overall survival according to tumor subtypes between MBC and FBC

Tumor subtype	Female (%)	Male (%)	cHR 95% CI	*P*	aHR 95% CI	*P*
**HoR-positive/HER2-negative**	95.8	93.8	1.496(1.186,1.888)	< 0.01	0.864(0.684,1.092)	0.221
**HoR-positive/HER2-positive**	95.0	85.8	2.763(1.773,4.305)	< 0.01	1.523(0.973,2.383)	0.066
**HoR-negative/HER2-positive**	91.6	83.3	2.186(0.545,8.761)	0.26	1.138(0.28,4.623)	0.857
**Triple negative**	89.0	77.4	3.113(1.482,6.539)	< 0.01	2.251(1.058,4.787)	0.035

Reference group for each model was ‘women’.

HER2 human epidermal growth factor receptor 2; HoR hormone receptor; cHR: crude hazard ratio; CI: confidence interval; aHR: adjusted hazard ratio (adjusted for age at diagnosis, race, grade, stage, tumor stage, node stage, distant metastasis, laterality and radiation).

### Impact of tumor subtype on BCSM in MBC and FBC

**Figure 2:**
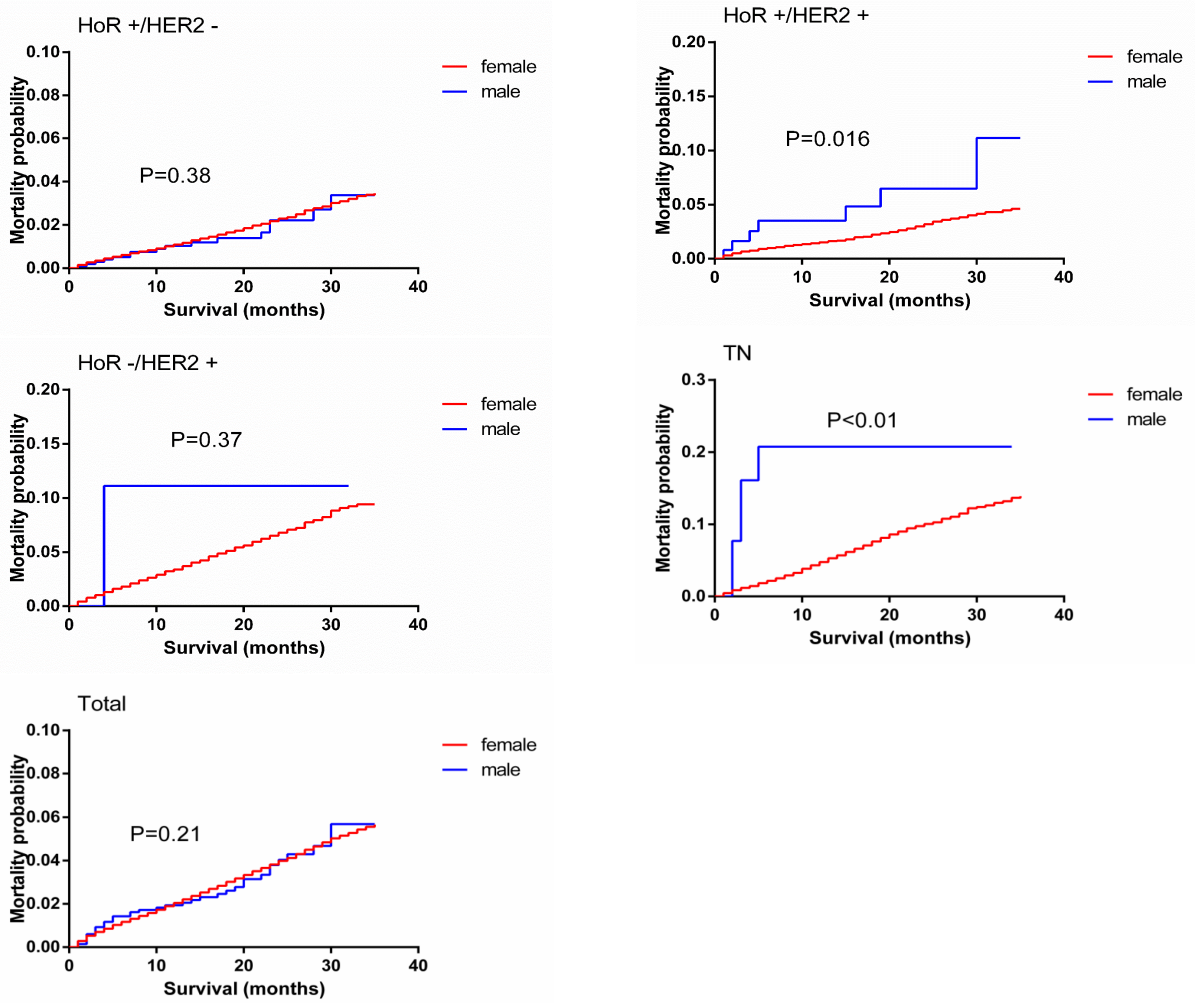
Breast-cancer-specific mortality curves of male matched with female breast cancer patients

A Kaplan-Meier analysis was used to determine BCSM in the groups based on sex. Individual survival curves for the four subgroups were generated (Figure 2). Unexpectedly, male and female patients exhibited similar survival rates; additionally, the stratified analysis showed similar survival rates for MBC and FBC in the HoR-positive/HER2-negative and HoR-negative/HER2-positive groups, whereas BCSM was increased for MBC compared to FBC in the HoR-positive/HER2-positive and TN groups (*p* = 0.016, *p* < 0.01).

Univariate and multivariate analyses were performed based on the Kaplan-Meier results. In the univariate analysis, age at diagnosis, race, tumor grade, laterality, tumor stage, tumor size, node stage, distant metastasis, HER2 status and history of radiation were significantly associated with BCSM (*p* < 0.05). The multivariate analysis was performed using the Cox regression model. All the factors mentioned above were identified as independent prognostic factors (Table 4), including age at diagnosis (50-64, HR = 1.355 (1.099, 1.672); ≥65, HR = 2.337 (1.897, 2.879)), race (black, HR = 1.346 (1.258, 1.441)), grade (moderately differentiated, HR = 1.848 (1.594, 2.142); poorly differentiated, HR = 3.868 (3.347, 4.471); undifferentiated, HR = 3.763 (2.833, 4.999)), tumor stage (II, HR = 1.20 (0.98, 1.48); III, HR = 1.72 (1.15, 2.61); IV, HR = 2.19 (1.88, 2.60)), tumor size (T2, HR = 2.919 (2.651, 3.214); T3, HR = 3.139 (2.797, 3.523); T4, HR = 6.984 (5.211, 8.963)), node stage (N1, HR = 1.692 (1.572, 1.822); N2, HR = 1.643 (1.48, 1.824); N3, HR = 1.553 (1.386, 1.74)), distant metastasis (M1, HR = 5.967 (5.599, 6.359)), laterality (bilateral, HR = 1.545 (1.37, 1.804)), HER2 status (positive, HR = 1.713 (1.658, 1.771)) and history of radiation (no, HR = 2.486 (2.223, 2.805)).

**Table 4:** Cox proportional hazards regression model analysis of breast cancer-specific mortality

Variables	Univariate analysis		Multivariate analysis	
	HR (95% CI)	*P*-value	HR (95% CI)	*P*-value
**Age at diagnosis, y**				
**<35**	Reference		Reference	
**35-49**	0.784(0.631,0.974)	0.028	1.082(0.871,1.344)	0.475
**50-64**	0.934(0.757,1.151)	0.021	1.355(1.099,1.672)	0.005
**≥65**	1.468(1.194,1.808)	< 0.001	2.337(1.897,2.879)	< 0.001
**Sex**	0.931(0.685,1.266)	0.21	0.721(0.53,1.081)	0.073
**Race**				
**white**	Reference		Reference	
**Black**	1.951(1.824,2.086)	< 0.001	1.346(1.258,1.441)	0.001
**Other**	0.706(0.629,0.792)	< 0.001	0.768(0.684,0.862)	< 0.001
**Unknown**	0.656(0.431,0.997)	0.048	0.477(0.313,0.981)	0.031
**Grade**				
**Well**	Reference		Reference	
**Moderately**	3.045(2.63,3.526)	< 0.001	1.848(1.594,2.142)	< 0.001
**Poorly**	8.836(7.677,10.171)	< 0.001	3.868(3.347,4.471)	< 0.001
**Undifferentiated**	13.544(10.238,17.917)	< 0.001	3.763(2.833,4.999)	< 0.001
**Unknown**	24.039(20.804,27.778)	< 0.001	3.35(2.871,3.91)	< 0.001
**Stage**				
**I**	Reference		Reference	
**II**	1.11 (0.90–1.36)	< 0.001	1.20 (0.98–1.48)	< 0.001
**III**	1.77 (1.18–2.67)	< 0.001	1.72 (1.15–2.61)	< 0.001
**IV**	2.19 (1.88–2.59)	< 0.001	2.19 (1.88–2.60)	< 0.001
**Unknown**				
**Tumor size**				
**T0/T1**	Reference		Reference	
**T2**	4.711(4.295,5.167)	< 0.001	2.919(2.651,3.214)	< 0.001
**T3**	8.751(7.874,9.726)	< 0.001	3.139(2.797,3.523)	< 0.001
**T4**	9.517(8.519,10.291)	< 0.001	6.984(5.211,8.963)	< 0.001
**NA**	3.73(2.944,4.794)	< 0.001	5.882(5.23,6.615)	< 0.001
**Node stage**				
**N0**	Reference		Reference	
**N1**	4.327(4.046,4.628)	< 0.001	1.692(1.572,1.822)	< 0.001
**N2**	4.802(4.354,5.297)	< 0.001	1.643(1.48,1.824)	< 0.001
**N3**	6.501(5.846,7.229)	< 0.001	1.553(1.386,1.74)	< 0.001
**NX**	12.241(10.525,14.099)	< 0.001	2.136(1.931,2.362)	< 0.001
**NA**	4.616(2.478,8.598)	< 0.001	1.806(1.428,1.515)	0.502
**Distant metastasis**				
**M0**	Reference		Reference	
**M1**	12.699(11.519,13.922)	< 0.001	5.967(5.599,6.359)	< 0.001
**Laterality**				
**Left**	Reference		Reference	
**Right**	0.957(0.906,1.011)	0.118	0.968(0.916,1.022)	0.239
**Paired**	1.493 (1.529,2.69)	0.551	1.399(1.227,1.595)	0.355
**Bilateral**	1.425(0.844,1.823)	0.025	1.545(1.37,1.804)	0.035
**Unknown**	1.263(0.709,1.645)	0.138	1.16(0.79,1.705)	0.449
**HER2**				
**Negative**	Reference		Reference	
**Positive**	1.344(1.243,1.452)	< 0.001	1.713(1.658,1.771)	< 0.001
**Borderline**	1.475(1.254,1.734)	< 0.001	1.075(0.914,1.264)	0.385
**Unknown**	1.350(1.296,1.415)	< 0.001	1.586(1.465,1.716)	< 0.001
**Radiotherapy**				
**Yes**	Reference		Reference	
**No**	2.454(2.027,2.972)	< 0.001	2.486(2.223,2.805)	< 0.001

Table 5 summarizes the BCSM identified for MBC and FBC *via* the subgroup analysis. The percentages of males and females who were dying from BC at the end of the study were compared (2.2% *vs*. 2.5%). The univariate analysis of BCSM according to tumor subtype showed significant differences between MBC and FBC, with higher mortality in HoR-positive/HER2-positive males than females with the same subtype (5.0% *vs*. 2.4%, HR = 2.144 (1.016, 4.526), *p* = 0.016). As expected for the TN subtype, BCSM was significantly greater in males (16.1%) than females (6.8%) at the end of the study (HR = 3.697 (1.536, 8.898), *p* < 0.01). The results of the multivariate analysis were somewhat inconsistent with those of the univariate analysis, as they showed that male patients were more likely to live than females with the HoR-positive/HER2-negative subtype (HR = 0.604 (0.369, 0.99), *p* = 0.045) and have poorer survival than females with the TN subtype (HR = 2.251 (1.058, 4.787), *p* = 0.013).

**Table 5:** Breast cancer-specific mortality according to tumor subtypes between MBC and FBC

Tumor subtype	Female (%)	Male (%)	cHR 95% CI	*P*	aHR 95% CI	*P*
**HoR-positive/HER2-negative**	1.7	1.2	0.881(0.539,1.441)	0.38	0.604(0.369,0.99)	0.045
**HoR-positive/HER2-positive**	2.4	5.0	2.144(1.016,4.526)	0.016	1.493(0.702,3.174)	0.297
**HoR-negative/HER2-positive**	4.9	8.3	1.827(0.257,13.004)	0.37	1.375(0.191,9.902)	0.868
**Triple negative**	6.8	16.1	3.697(1.536,8.898)	< 0.01	3.125(1.273,7.668)	0.013

Reference group for each model was ‘women’.

HER2 human epidermal growth factor receptor 2; HoR hormone receptor; cHR: crude hazard ratio; CI: confidence interval; aHR: adjusted hazard ratio (adjusted for age at diagnosis, race, grade, stage, tumor stage, node stage, distant metastasis, laterality and radiation).

## DISCUSSION

MBC is a rare disease, and its etiology is neither completely characterized nor fully understood. In contrast with FBC, there have been no prospective randomized studies allowing the biological characteristics of MBC or clinical and therapeutic approaches to be delineated with any certainty. The current knowledge base regarding MBC is derived from the data collected in retrospective studies.

Given that MBC is rare, there are no routine screening guidelines for men, which would delay the diagnosis [3, 14]. Consistent with the findings of previous studies, in this study, males tended to be older than females when diagnosed [3, 15]. The prevalence of comorbidities increased with age, which likely contributed to the decreased OS in MBC than FBC. Donegan et al. [16] reported that the high rate of post-treatment mortality from comorbidities, such as heart disease or other cancer types, was a major contributor to the poor survival observed in MBC. In our population-based comparison, MBC had more advanced stages (stages II-IV), lower grades, larger sizes, more lymph node and distant metastases than FBC. While MBC patients had poorer OS, there was no disparity in BCSM. The univariate analysis showed that survival was generally poorer in TN MBC than TN FBC in terms of both OS and BCSM, while for the HoR-positive/HER2-negative subtype, MBC had a longer survival period than FBC in terms of BCSM.

Given that MBC is rare, there are no standard treatments guidelines for men, which could cause disparities in survival. Several studies have identified a higher frequency of HoR positivity in MBC than FBC, suggesting that endocrine therapy could be useful in this population. Some studies reported that endocrine therapy was associated with improved survival in men [17–19], while other studies have suggested that estrogen receptor (ER)-positive tumors in males and females do not respond to tamoxifen therapy in the same manner. As a result, HoR positivity was not indicative of a good prognosis in MBC [20, 21]. In view of the difference in BCMS observed in the HoR-positive/HER2-negative subtype, endocrine therapy might be as effective in MBC as in FBC. MBC is a hormone-driven disease that often leads to androgen receptor (AR) expression, with AR expression rates reportedly ranging from 34-95% in MBC [22–24]. Compared to FBC, a larger fraction of ER+ MBCs seem to be more connected with AR- than ER-associated signaling [25], suggesting that coordinated ER and AR expression could be relevant to MBC biology. Several subsequent reports have suggested that gonadotropin-releasing hormone analogues in combination with aromatase inhibitors could improve the survival outcome of MBC. Further investigation could lead to the discovery of anti-hormonal therapies based on new intrinsic subtypes, which could improve the clinical management of MBC.

Conflicting data have been reported regarding HER2 status in males as a prognostic indicator. Several studies have shown a lower expression or a lack of prognostic significance of HER2 in MBC compared with FBC, but some studies have instead demonstrated HER2 over expression in MBC [26–29]. While our analysis showed that HER2 status was a prognostic indicator for OS, there was no difference in its prognostic effect between male and female survival. Thus, despite the similar expression of biomarkers, MBC might yet be biologically different from FBC in other ways.

Triple-negative breast cancers (TNBCs) are a heterogeneous group of tumors with higher relapse rates and shorter OS in the metastatic setting. As expected, in the TN subtype, survival was significantly different between the sexes. From a genetic perspective, tumors arising in carriers of BReast CAncer (BRCA) genes are similar to TNBCs in many ways. After BRCA2 was identified, BRCA2 mutations were found in families affected by both FBC and MBC. BRCA2 mutations have been found to significantly increase the risk of MBC [30] and identified as independent, adverse prognostic factors in MBC [31]. Additionally, approximately 20% of patients with MBC were found to have a family history of BC [32]. Other genetic alterations that have been connected with the onset of MBC involve PALB2, CYP17, CHEK2, and RAD51B [33]. A new class of agents has shown promising activity in BRCA-deficient BC patients, such as DNA-damaging cytotoxic agents (e.g., cisplatin) and targeting poly(ADP-ribose) polymerase inhibitors (e.g., olaparib) [34, 35]. The association observed between MBC and BRCA-deficient disease suggests the potential therapeutic applicability of olaparib, cisplatin and other related agents in this condition, although this clearly requires further clinical validation.

The molecular characterization of MBC has offered insights into potential therapeutic strategies. In one report [36], immunohistochemistry analyses showed higher expression levels of homeobox D10 (HOXD10) and vascular endothelial growth factor (VEGF) in MBC than FBC specimens. HOXD10 is involved in cell migration and extracellular matrix remodeling. Meanwhile, VEGF is a driver of tumor-related angiogenesis; several agents (e.g., bevacizumab) can antagonize VEGF-mediated signaling and improve progression-free survival when combined with cytotoxic chemotherapy for treating metastatic FBC [37]. Given these data, the exploration of VEGF-directed therapies for MBC might be warranted. In a series of 30 patients with male gynecomastia and 30 patients with MBC, prolactin receptor expression was significantly higher in the MBC patients than in the patients with gynecomastia [38]. Compounds antagonizing the prolactin receptor have been shown to augment the activity of doxorubicin and paclitaxel in cellular models; as such, this approach might be clinically useful for treating MBC [39].

Our study has some limitations. Because SEER collected information about HER2 status since 2010, our analysis had a short follow-up period, which might affect the interpretation of our results. We did not have information for this cohort regarding systemic treatments, such as surgery and chemotherapy, which might contribute to some of the differences observed in survival according to tumor subtype. However, the male patients in this study were less likely to receive radiation than females.

In summary, we identified clear differences between MBC and FBC in both OS and BCSM in the TN subtype, which warrant further investigation. Future translational studies require prospective validation and should focus on the tumor biology and treatment efficacy of MBC. However, our study has laid a foundation for using tumor subtype differences between the sexes to develop and evaluate personalized therapies for MBC in clinical trials.

## MATERIALS AND METHODS

### Data source and study design

We obtained data from the National Cancer Institute's SEER program between 2010 and 2012. SEER started collecting information on HER2 status in 2010. Therefore, we used that year as the starting point for our study. We extracted data for all cases of invasive BC diagnosed between 2010 and 2012. We selected cases with known HoR and HER2 statuses.

The demographic variables included age at diagnosis (< 35, 35-49, 50-64, > 65 years) and race (white, black, other). The cancer characteristics included stage (I, II, III, IV, unknown), grade (well differentiated, moderately differentiated, poorly differentiated, undifferentiated, unknown), T stage (T0/T1, T2, T3, T4, NA), N stage (N0, N1, N2, N3, NX, NA), distant metastasis (M0, M1, NA), laterality (right, left, paired, bilateral, unknown), and HoR and HER2 status (positive, negative, borderline, unknown). The treatment characteristics included receipt of radiation therapy (no, yes, unknown). Tumor subtypes were classified as HoR-positive/HER2-negative, HoR-positive/HER2-positive, HoR-negative/HER2-positive, and triple-negative (TN) subtypes according to the breast subtype variable.

The two main outcomes in our study were OS and BCSM. Vitality status was recorded as “alive” or “dead” in the SEER dataset. Survival time (in months) was calculated for each patient using the “Completed Months of Follow-up” option in the SEER database. OS was determined by comparing males and females who were alive at the end of the study period or who were alive at their last follow-up. BCSM was determined by comparing males and females whose cause of death was due to BC with males and females who were alive at the end of the study period, had died due to other causes, or who were alive at their last follow-up. Cases without survival times were classified as unknown and removed from the study.

### Statistical analysis

Patient demographics and cancer- and treatment-related characteristics were compared between females and males using Chi square or Fisher's exact tests as appropriate. Within each variable, patients with unknown data were excluded from the comparative analysis. A matched subgroup analysis was performed. Survival probabilities for OS and BCSM were estimated using the Kaplan-Meier method, and variables were compared using the log-rank test in the subgroups. Univariate and multivariate Cox proportional hazard regressions were used to obtain HRs and their respective 95% confidence intervals and show the strength of the estimated relative risk; these approaches were applied to model the relationship between potential covariates and either OS or BCSM. All statistical analyses were performed using SPSS 19.0 (IBM Corporation, Armonk, NY), and all charts of survival probabilities were prepared using GraphPad Prism 6.0. Two-sided p values less than 0.05 were considered statistically significant.
